# Laryngeal cryptococcus: a rare cause of hoarseness in renal allograft recipient

**Published:** 2015-11-15

**Authors:** Jashan Sandhu, Jasvinder Singh Sandhu, Harpreet Kaur Puri, Manish Munjal

**Affiliations:** Department of Nephrology, Dayanand Medical College and Hospital, Ludhiana, Punjab, India

**Keywords:** Hoarseness, Laryngeal cryptococcus, Renal allograft recipient

## Abstract

Cryptococcosis commonly involves central nervous system and lungs in organ transplant recipients. Isolated laryngeal infection is extremely rare. We report a rare case of cryptococcus in a renal allograft recipient that clinically presented with hoarseness of voice and mimicked laryngeal carcinoma on examination.

**Implication for health policy/practice/research/medical education:**

Cryptococcosis commonly involves central nervous system and lungs in organ transplant recipients. Isolated laryngeal infection is extremely rare. This is a rare case of cryptococcus in a renal allograft recipient that clinically presented with hoarseness of voice and mimicked laryngeal carcinoma on examination.

## Introduction

*Cryptococcus neoformans* is ubiquitous yeast that is commonly found in soil, especially in association with pigeon droppings (1). Infection usually occurs via inhalation of soil aerosols. The clinical manifestations of cryptococcal disease depend on the immune state of the host. In renal allograft recipients cryptococcus can cause primary pulmonary infections or disseminate and cause infections of central nervous system, meninges, skin and bone (2). Laryngeal cryptococcus is rare (3,4).

## Case Presentation

A 30-year-old non-smoker male, carpenter by occupation was detected to have end-stage renal disease due to chronic glomerulonephritis in 2006. He remained on intermittent hemodialysis till he received a renal allograft from his 50 years old mother in August 2008. He was not given induction therapy. He received triple immunosuppression that included oral prednisolone, mycophenolate mofetil and tacrolimus in therapeutic doses. Due to financial constraints, mycophenolate mofetil was replaced with oral azathioprine 100 mg a day after 3 months of renal transplantation. He maintained normal graft function. Four years after transplant, he presented with hoarseness of voice of 2 months duration. This was insidious in onset, progressively increasing and was not associated with neck pain, voice fatigue and cough. There was no relief with voice rest. There was no history of fever, hemoptysis and weight loss.

His general physical and systemic examination was essentially unremarkable. There was no tenderness or bruit over the graft kidney. The local examination of the nose, throat and ear did not show any gross abnormality. Flexible fiberoptic laryngoscopy showed an exophytic growth on the vocal process of the left arytenoid, extending anteriorly till the anterior commissure. The growth was excised under general anaesthesia and the base fulgurated. The tissue was sent for histopathological and microbiological examination, to evaluate for neoplastic and fungal pathology.

**Figure 1:**
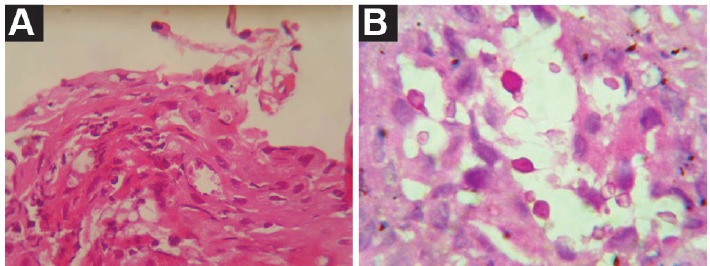
(A) H&E stain showing the cryptococcus (×400). (B) PAS stain highlighting the cryptococcus (×400).

On investigations, the complete blood count (CBC) showed mild anemia with hemoglobin of 12.5 gm/dl and normal total and differential leukocyte and platelet counts. Urine examination showed absence of proteinuria and hematuria. The blood biochemistry revealed blood urea and serum creatinine of 40 mg/dl and 1.5 mg/dl respectively. His serology tests for hepatitis B surface antigen and antibodies to hepatitis C virus, Human immunodeficiency virus and cytomegalovirus were negative. Chest x-ray was normal. Biopsy from laryngeal mass lesion exhibited a polypoidal appearance lined by stratified squamous epithelium underneath which sheets of histiocytes containing numerous spores of cryptococcus of variable sizes were seen (Figure 1A). The PAS stain highlighted the fungus spores (Figure 1B). While, clinically there were no signs and symptoms of meningitis, the cerebrospinal fluid (CSF) examination was done that was negative for cryptococcal meningitis. Patient was treated with oral fluconazole 400 mg per day for 6 months and recovered completely. Since fluconazole has interaction with calcineurin inhibitors, the dose of tacrolimus was decreased to 1 mg twice a day maintaining the blood levels in the therapeutic range.

## Discussion

There are numerous causes of hoarseness of voice. Most of these can be divided into three broad categories: inflammatory lesions, benign proliferative lesions, and malignant tumours.

Although viral laryngitis is the most common infectious cause of hoarseness, primary mycotic laryngeal infections have been described, most of them existing in the tissues as yeasts. Relative to other fungal infections of the larynx, *Cryptococcus neoformans* laryngitis is uncommon (5). *Cryptococcus neoformans* is identified in tissue sections as eosinophilic or lightly basophilic yeasts that vary in size from 2 to 20 mm. These yeasts possess capsules that stain positively with mucicarmine and cell walls that stain positively with methenamine silver. In immunocompetent patients, *Cryptococcus neoformans* usually elicits a granulomatous reaction whereas in an anergic patient, the host reaction may be greatly diminished (6).

Common characteristics of the previously reported cases of laryngeal cryptococcus include history of hoarseness and cough, erythema and edema of the vocal cord, verrucous/warty type gross appearance, and pseudoepitheliomatous hyperplasia of the laryngeal mucosa seen on microscopic examination. Laryngeal involvement is either via a hematogenous route with spread from a primary focus such as the lung or from direct implantation of inhaled aerosolized organisms (3,7).

Mycotic laryngitis mimicking a malignant process as observed in our case has also been described in reports of other mycotic laryngeal infections (8,9). Given the verrucous appearance of a fungal infection in the larynx, the potential to confuse a mycotic infection with laryngeal carcinoma can be high. The clinical evaluation of hoarseness, including history, physical examination, and direct visualization of the larynx will often provide enough evidence to lead to a presumptive diagnosis. Our case emphasizes the need for biopsy confirmation, since other pathologic entities including fungal infections may clinically mimic a malignant process

Cryptococcosis has been documented in an average of 2.8% of solid-organ transplant recipients. The median time for disease onset is 21 months after transplantation (2). Patients receiving a calcineurin-inhibitor-based regimen were less likely to have disseminated disease and more likely to have cryptococcus limited to the lungs (10).

There are no randomized, prospective trials of antifungal treatment of solid organ transplant recipients. Infectious diseases society of America in 2010 update has made explicit recommendations for the management of cryptococcal disease (11). For meningoencephalitis or disseminated cryptococcus in organ transplant recipients, induction therapy, amphotericin B deoxycholate (0.7 to 1.0 mg/kg/day intravenously) plus flucytosine (100 mg/kg/day in 4 divided doses) for 2 weeks is recommended for induction therapy. Lipid formulations of amphotericin B including liposomal (3-4 mg/kg/day) or amphotericin B lipid complex (5 mg/kg/day) could be substituted for amphotericin B and are favoured in organ transplant recipients receiving calcineurin inhibitors. This should be followed by a consolidation phase with oral fluconazole (400 to 800 mg/day) for 8 weeks and finally maintenance phase using fluconazole 200 to 400 mg/day for 6-12 months. For mild to moderate pulmonary disease, fluconazole 400 mg (6 mg/kg) per day for 6-12 months is recommended.

Since fluconazole increases the levels of calcineurin inhibitors, immunosuppressive management should include sequential or step-wise reduction of calcineurin inhibitor, with consideration of lowering the corticosteroid dose first (12). Rapid reductions of immunosuppressives can have untoward side effects such as development of graft rejection and/or immune reconstitution inflammatory syndrome (13).

## Conclusion

Cryptococcosis commonly involves central nervous system and lungs in organ transplant recipients. Isolated laryngeal infection is extremely rare. In this paper we described a rare case of cryptococcus in a renal allograft recipient that clinically presented with hoarseness of voice and mimicked laryngeal carcinoma on examination. Patient was treated with oral fluconazole 400 mg per day for six months and recovered completely. However, routine antifungal prophylaxis for cryptococcosis is not recommended in renal allograft recipients since there are no evidence-based studies to support this and there is no precision in identifying specific high-risk group.

## Authors’ contribution

All authors contributed to the manuscript equally.

## Conflicts of interest

The authors declared no competing interests.

## Ethical considerations

Ethical issues (including plagiarism, data fabrication, double publication) have been completely observed by the author.

## Funding/Support

None.
